# Absence of peripapillary retinal nerve-fiber–layer thinning in combined antiretroviral therapy-treated, well-sustained aviremic persons living with HIV

**DOI:** 10.1371/journal.pone.0229977

**Published:** 2020-03-10

**Authors:** Cedric Lamirel, Nadia Valin, Julien Savatovsky, François-Xavier Lescure, Anne-Sophie Alonso, Philippe Girard, Jean-Paul Vincensini, Pierre-Marie Girard, Laurence Salomon, Isabelle Cochereau, Antoine Moulignier

**Affiliations:** 1 Département d’Ophtalmologie, Fondation Adolphe de Rothschild, Paris, France; 2 Service d’Ophtalmologie, APHP, Hôpital Bichat–Claude-Bernard, Paris, France; 3 Service des Maladies Infectieuses et Tropicales, APHP, Hôpital Saint-Antoine, Paris, France; 4 Service d’Imagerie Médicale, Fondation Adolphe de Rothschild, Paris, France; 5 Service des Maladies Infectieuses et Tropicales, APHP, Hôpital Bichat–Claude-Bernard Paris, Paris, France; 6 Unité de Recherche Clinique, Fondation Adolphe de Rothschild, Paris, France; 7 Service de Pneumologie, Institut Mutualiste Montsouris, Paris, France; 8 Sorbonne Paris Cité, Université Paris Diderot, Paris, France; 9 Service de Neurologie, Fondation Adolphe de Rothschild, Paris, France; University of Florida, UNITED STATES

## Abstract

**Purpose:**

To compare peripapillary retinal nerve-fiber–layer (pRNFL) thickness, total retina macular volume, and ganglion-cell-layer (GCL) macular volume and thickness between persons living with HIV (PLHIVs) with well-controlled infections and good immune recovery, and sex- and age-matched HIV-uninfected controls (HUCs).

**Methods:**

This prospective cross-sectional study (www.clinicaltrials.gov identifier: NCT02003989) included 56 PLHIVs, infected for ≥10 [median 20.2] years and with sustained plasma HIV-load suppression on combined antiretroviral therapy (cART) for ≥5 years, and 56 matched HUCs. Participants underwent spectral-domain optical coherence tomography (SD-OCT) with thorough ophthalmological examinations and brain magnetic resonance imaging (MRI). Their overall and quadrant pRNFL thicknesses, total macular volumes, and GCL macular volumes and thicknesses were compared. Cerebral small-vessel diseases (CSVD) complied with STRIVE criteria.

**Results:**

Median [interquartile range, IQR] ages of PLHIVs and HUCs, respectively, were 52 [46–60] and 52 [44–60] years. Median [IQR] PLHIVs’ nadir CD4+ T-cell count and current CD4/CD8 T-cell ratio were 249/μL [158–350] and 0.95 [0.67–1.10], respectively; HIV-seropositivity duration was 20.2 [15.9–24.5] years; cART duration was 16.8 [12.6–18.6] years; and aviremia duration was 11.4 [7.8–13.6] years. No significant between-group pRNFL thickness, total macular volume, macular GCL-volume and -thickness differences were found. MRI-detected CSVD in 21 (38%) PLHIVs and 14 (25%) HUCs was associated with overall thinner pRNFLs, and smaller total retina and GCL macular volumes, independently of HIV status.

**Conclusions:**

SD-OCT could not detect pRNFL thinning or macular GCL-volume reduction in well-sustained, aviremic, cART-treated PLHIVs who achieved good immune recovery. However, CSVD was associated with thinner pRNFLs and GCLs, independently of HIV status.

## Introduction

Combined antiretroviral therapy (cART) ensures human immunodeficiency virus (HIV) suppression and immunological recovery in a majority of persons living with HIV (PLHIVs), dramatically improving life expectancy [1,2]. As a consequence, cART-treated PLHIVs are exposed to chronic HIV-infection that may be deleterious to neural tissues [3,4]. Hence, despite well-sustained immunovirological control on cART, subtle structural and functional retinal abnormalities, described as HIV-associated neuroretinal disorder (HIV–NRD) and milder forms of HIV-associated neurocognitive disorders (HAND) are still frequent in PLHIVs [5,6]. In addition, aging PLHIVs’ life expectancy persistently lags behind that of the general population, predominantly because of their heightened risk for age-related comorbidities, to which they might be more vulnerable [7,8]. Among those age-related comorbidities, magnetic resonance imaging (MRI)-detected [9] cerebral small-vessel disease (CSVD) prevalence is doubled in cART-treated, immunovirologically well-controlled, middle-aged PLHIVs compared to age-matched HIV-uninfected individuals [10]. The best-known MRI characteristics of CSVD are white-matter hyperintensities (WMHs) of presumed vascular origin, silent brain infarcts and cerebral microbleeds [11]. To better characterize and differentiate CSVD-surrogate WMHs from WMHs of other origins, the STandards for ReportIng Vascular changes on Euroimaging (STRIVE) criteria, developed to standardize reading of CSVD neuroimages, were applied [9]. The results of several studies [12–15] documented the cognitive impact of CSVD-surrogate WMHs on cART-treated PLHIVs with long-term virus suppression, leading to the recent paradigm of vascular-driven, milder HAND forms [16].

The concept of the retina being an anatomical and functional central nervous system surrogate is increasingly recognized [17]. Notably, cerebral and retinal arterioles share similar anatomy, physiology and embryology, and evidence supports an association between retinal vessel changes and CSVD [18]. Optical coherence tomography (OCT) is an in situ micrometer-scale imaging technique that closely correlates with histological retinal structures [19,20]. Indeed, OCT accurately and reproducibly measures the peripapillary retinal nerve-fiber–layer (pRNFL) thickness that reflects the number of ganglion-cell axons leaving the retina to form the optic nerve. OCT can also evaluate the thickness of the ganglion-cell–layer (GCL) macula that contains mostly ganglion-cell bodies.

Spectral-domain (SD)-OCT detected significant pRNFL thinning in severely immunodeficient PLHIVs (i.e., CD4+ T-cell count <100/μL) [21–23]. In PLHIVs, GCL thinning has been associated with HAND [24]. However, those findings were heterogeneous, and all studies were hampered by the absence of brain and orbit MRI to exclude optic neuropathy or CSVD (Table 1). Indeed, pRNFL thinning has been associated with optic neuropathies [25], and CSVD-surrogate WMHs in the general population [26,27] and HIV-infected children [28].

**Table 1:** Reported peripapillary retinal nerve-fiber–layer (pRNFL) thicknesses in PLHIV or HUC participants.

[Ref] Sample	Age (y)	Years HIV+	Years on cART	CD4+ T-cell (cells/μL)		CD4/CD8 T-cell ratio	Current plVL log_10_	% plVL undetectable	Years of aviremia	OCT	pRNFL thickness (μm)				
				Nadir	Current						Overall^a^	Superior	Inferior	Nasal	Temporal
[5]															
93 HIV+	53.5 {45–76}	14.5 {1–27}	12 {1–21}	180 {0–620}	595 {320–1110}	0.75 {0.29–4.13}	1.6 {1.6–1.94}	98.9%	10.2 {0–15.1}	SD	102.0±11.3	125.5±20.2	131.1±16.8	78.5±15.3	72.9±13.5
63 HIV–	52 {45–80}										100.0±10.1	125.4±17.3	128.1±15.8	74.2±16.0	72.4±12.5
[21]															
25 HIV+	42.68±1.61	7.2±0.8	6.4±0.9	301.08±31.11	502±41.36	NR	NR	NR	NR	TD	107.3±11.0**	133.9±21.1**	138.0±16.5**	87.4±17.0	69.7±13.0
26 HIV+	43.54±1.51	8.4±1.3	5.9±1.1	28.31±4.67	219.96±49.18	NR	NR	NR	NR		96.1±21.3*	119.4±32.6	122.9±32.2*	76.7±27.6*	65.7±12.8
22 HIV–	43.27±2.59										107.2±12.7	129.4±16.7	138.6±17.6	89.4±22.3	71.4±12.4
[22]															
12 HIV+	NR	NR	NR	>100	NR	NR	NR	NR	NR	TD	97.2±12.8	NR	NR	NR	NR
10 HIV+	NR	NR	NR	≤100	NR	NR	NR	NR	NR		74.6±17.4**	NR	NR	NR	NR
[23]															
18 HIV+	41.77±8.15	NR	NR	>100	NR	NR	NR	NR	NR	TD	103.3±9.3	127.5±17.6	138.6±14.3	74.6±15.4	73.2±14.7
25 HIV+	41.64±6.61	NR	NR	≤100	NR	NR	NR	NR	NR		90.1±12.5*	110.3±20.7*	112.4±22.9*	72.2±12.4	65.2±16.9*
22 HIV–	38.22±9.39										103.3±8.5	122.7±12.7	137.2±17.5	78.3±15.1	75.9±17.7
[24]															
69 HIV+ NCI	}53.1±7.3	}14.5±8.6	}NR	}171.8±145.9	}676.1±306	}NR	}<1.5	}NR	}NR	SD	97.6±8.6	NR	NR	NR	72.1±9.6
64 HIV+ CI											95.2±13.8	NR	NR	NR	68.5±14.1
70 HIV–	51.6±7.5										98.4±9.8	NR	NR	NR	71.3±8.7
[42]															
33 HIV+	12.1 [11.5–15.8]	NR	10.7 {7.1–14.4}	NR	760 [580–950]	NR	NR	82%	NR	SD	112.1±15.8	NR	NR	NR	NR
36 HIV–	13.7 [12.2–15.8]										112.2±9.5	NR	NR	NR	NR
[43]															
225 HIV+	41.2±0.5	NR	4.7 [2.8–6.2]	NR	NR	NR	NR	NR	NR	SD	109.7±12.0	135.1±21.0	135.6±21.0	91.1±22.5	73.1±13.5
203 HIV–	41.9±0.6										108.7±12.8	132.2±21.4	137.9±21.4	88.4±21.4	72.5±12.8
[45]															
19 HIV+	53.9 {44–73}	17.0	NR	>200	NR	NR	≤1.7	NR	NR	SD	92.7±14.4	NR	NR	76.3±18.3	64.2±12.2
28 HIV+	55.1 {41–85}	18.2	NR	<200	NR	NR	≤1.7	NR	NR		91.0±11.1	NR	NR	72.2±18.5	65.7±15.3
57 HIV–	56.2 {28–84}										94.6±9.8	NR	NR	70.7±16.6	69.7±12.1
[46]															
51 HIV+	43.12±7.80	7.8±5.5	6.2±5.0	162.02±175.69	355.48±267.69	NR	NR	NR	NR	TD	99.9±13.7	NR	NR	NR	NR
22 HIV–	43.27±12.15										105.2±9.0				
[60]															
45 HIV+	37.3 {17–75}	NR	NR	NR	426 {36–954}		5.2 {1–6.1}	55.6%		SD	100.5±9.5	122.4	131.2	78.5 ±15.0	69.6±9.1
47 HIV–	39.4 {20–38}										102.0±8.9	127.5	131.8	77.1±14.7	72.4±9.4
Our results															
56 HIV+	51.5 [45.5–59.5]	20.2 [15.9–24.5]	16.8 [12.6–18.6]	249 [158–350]	691 [526–1053]	0.95 [0.67–1.10]	1.0 [1.0–1.0]	100%	11.4 [7.8–13.6]	SD	99.5±9.6	121.4±14.8	128.9±14.5	75.9±15.2	70.7±11.5
56 HIV–	52.0 [44.0–60.0]										99.6±8.3	123.8±14.8	130.2±14.5	75.6±15.2	68.9±11.5

Values are expressed as mean±standard deviation, or median [interquartile range] or {range}.

^a^ Overall values are the averages each individual 4 quadrants.

PLHIVs: human immunodeficiency virus-infected participants; HUCs: HIV-uninfected controls; OCT: optical coherence tomography; TD: time-domain; SD: spectral-domain; plVL: plasma HIV load; IQR: interquartile range; NR: not reported; NCI: no cognitive impairment. CI: cognitive impairment.

*Statistically significant (p<0.05) difference between HIV+ subjects and HIV–controls.

**Statistically significant (p<0.05) difference between high and low CD4+ T-cell nadirs for HIV+ patients.

We undertook this concurrent cohort study to investigate pRNFL and GCL thicknesses in PLHIVs with well-sustained, cART-controlled, immunovirological parameters and HIV-uninfected controls (HUCs). Because we wanted to examine the role of chronic HIV infection itself, we selected PLHIVs with cART-sustained, immunovirological control for at least 5 years, without hepatitis C virus (HCV) infection, past or ongoing acquired immune deficiency syndrome (AIDS)-defining neurological events (ADNEs), and/or alcohol or illicit drug abuse.

## Methods

### Ethics approval

This study, approved by the CPP Île-de-France VI Ethics Committee, adhered to the tenets of the Declaration of Helsinki. Written informed consent was obtained from all participants.

### Study population

In this cross-sectional study (NCT02003989), we prospectively included PLHIVs followed in two Infectious Diseases Departments in University Hospitals, caring for about 8,000 PLHIVs in the Paris area (France). Inclusion criteria were: (1) HIV seropositivity known for ≥10 years; sustained CD4+ T-cell count ≥350/μL and plasma HIV load (plVL) <20 copies/mL for ≥5 years on cART. plVL was quantified using the Amplicor monitor assay (Cobas 2.0, Roche Diagnostics, Basel, Switzerland), which has a lower detection limit of 20 HIV-1 RNA copies/mL. Exclusion criteria were: (1) transient low-level viremias ≥20 but ≤200 copies/mL (viral blip) once within the previous 5 years; (2) history/concomitant ocular trauma or diseases; (3) family history of glaucoma; (4) prior/current treatment with drugs associated with toxic optic neuropathy or retinopathy; (5) prior/current neurological/psychiatric disorders, including ADNE; (6) prior/current diabetes mellitus; (7) prior/current alcohol or illicit substance abuse (with the exception of occasional cannabis use); (8) HCV infection. Cognitive decay was not sought prior to inclusion and was not an exclusion criterion.

Age (±5 years)- and sex-matched (1:1) HUCs were selected on a voluntary basis; exclusion criteria were the same as for PLHIVs. The absence of HIV infection was confirmed by ELISA or rapid HIV test.

All participants underwent the same comprehensive neurological and ophthalmological examinations. All SD-OCT were obtained with a Spectralis OCT, which generates four sectoral pRNFL thicknesses and an overall value that is the average of the four quadrants, using the new Nsite Axonal Analytics software (Heidelberg Engineering GmbH, Heidelberg, Germany). Overall and sectoral pRNFL thicknesses were recorded for each eye. The total retinal macular volume was calculated with the Heidelberg software using the Early Treatment for Diabetic Retinopathy Study (ETDRS) grid. We used the Iowa Reference Algorithm to segment the GCL and calculated the GCL thickness for each of the 9 ETDRS-grid subfields and the total GCL macular volume within the ETDRS grid (S1 Fig) [29–31]. Cognition was assessed using the Montreal Cognitive Assessment (MoCA), known to be an adequate clinical and ecological screening tool for PLHIVs [32,33]. 3-Tesla brain MRI and orbit images (Philips Healthcare, Best, The Netherlands) were analyzed by one neuroradiologist (J.S.) blinded to all parameters. CSVD was defined according to the Standards for Reporting Vascular Changes on Neuroimaging (STRIVE) Criteria [9].

### Main outcome measure and estimated number of participants

The main outcome measure was the overall pRNFL thickness. Previous studies found a standard deviation of ~9 μm for it with the OCT machine used herein [34]. With that standard deviation, a unilateral 5% alpha-risk and 95% power, we estimated that 50 participants in each group would be sufficient to detect ≥7-μm pRNFL thinning in PLHIVs compared to HUCs.

### Statistical analyses

Only one eye of each participant was randomly selected for analysis. The non-parametric Mann-Whitney U-test and chi^2^ or Fisher’s exact test were used as appropriate to compare groups. Analysis of variance (ANOVA) of repeated measures was used to detect differences among the different pRNFL quadrants and among the different GCL ETDRS-grid subfields.

Linear-regression models were used to test associations between OCT measurements (outcome variables: overall and temporal pRNFL thicknesses, total retinal macular volumes, GCL macular volumes) and visual function measurements, axial length and age (predictor variables). HIV status was included in all these linear-regression models to test a possible HIV effect on an association.

For the PLHIV group, associations between overall and temporal pRNFL thicknesses, total retinal macular volumes, GCL macular volumes (outcome variables) and duration of HIV infection (predictor variable) were tested with a multivariate model including age as a covariate. Univariate regression analyses were used to identify associations between PLHIVs’ overall and temporal pRNFL thicknesses, total retinal macular volumes or GCL macular volumes (outcome variables) and CD4+ T-cell nadirs (predictor variable).

Significance was defined as p<0.05. All analyses were computed with Statistica software (Statsoft, Inc, Maison Alfort, France) and no statistical correction was made for multiple comparisons.

## Results

Among 71 PLHIVs initially included, 15 were secondarily excluded because of bilateral glaucoma (n = 1), bilateral high ametropia (n = 6), previous bilateral ocular surgery (n = 3), alcoholism (n = 1), diabetes (n = 2), HCV infection (n = 1) or missing MRI (n = 1). Among 65 HUCs initially included, nine were secondarily excluded because of bilateral high ametropia (n = 4), bilateral glaucoma (n = 1), diabetes (n = 1), HCV infection (n = 1), missing MRI (n = 1) or MRI-detected meningioma (n = 1). A total of 56 PLHIV-eyes and 56 HUC-eyes were included in the statistical analyses. Participants’ characteristics are summarized in Table 2.

**Table 2:** Epidemiological, clinical, biological and radiological characteristics of PLHIV and HUC participants.

Characteristic	PLHIVs (n = 56)	HUCs (n = 56)	p value
Demographic			
Age at enrollment, y	51.5 [45.5–59.5]^a^	52.0 [44.0–60.0]	0.96^b^
Age at HIV diagnosis, y	32.8 [26.6–39.9]	–	
Caucasian, n (%)	56 (100%)	56 (100%)	
Male, n (%)	49 (87.5)	49 (87.5)	1.00^c^
Education ≥12 y, n (%)	44 (78.6)	43 (76.8)	0.82^b^
MoCA score	27 [25–29]	28 [26–29]	0.15^c^
Smoking status, n (%)			
Current smoker	14 (25)	21 (37.5)	<0.05^c^
Former smoker	20 (35.7)	8 (14.3)	
Never smoker	22 (39.3)	25 (44.6)	
Alcohol consumption			0.51^c^
Never, n (%)	6 (10.7)	8 (14.3)	
≤3 glasses/week, n (%)	50 (89.3)	44 (78.6)	
≤2 glasses/day, n (%)	0	2 (3.4)	
Comorbidities			
Controlled hypertension, n (%)	14 (25)	4 (7.1)	0.01^c^
Cardiovascular disease, n (%)	8 (14.3)	0	<0.01^d^
Renal disease	0	0	
Body mass index (kg/height^2^)	22.5 [20.8–25.0]	NR	
Frailty status ≥3	0	0	
Brain and orbit MRI			
Cerebral small-vessel disease, n (%)	21 (37.5)	14 (25)	0.15^c^
White matter hyperintensities, n (%)	21 (37.5)	12 (21.4)	0.06^c^
Cerebral microbleeds, n (%)	2 (3.6)	4 (7.1)	0.68^d^
Silent brain infarcts, n (%)	1 (1.8)	1 (1.8)	1.00^d^
HIV-transmission route			
Homosexual/bisexual, n (%)	42 (75)	–	
Heterosexual, n (%)	10 (17.9)		
IVDU, n (%)	3 (5.4)	–	
Unknown, n (%)	1 (1.8)	–	
CDC stages A/B/C, n (%)	31 (55)/12 (22)/13 (23)	–	
HIV-seropositivity duration, y	20.2 [15.9–24.5]	–	
AIDS duration, y (n = 10)	17.1 [7.5–23.9]	–	
Antiretroviral treatment			
Duration, y	16.8 [12.6–18.6]	–	
Treatment regimens, n	4 [3–8]	–	
Immunology/virology			
Nadir CD4+ T-cell count (cells/μL)	249 [158–350]	–	
Current CD4+ T-cell count (cells/μL)	691 [526–1053]	–	
Current CD8+ T-cell count (cells/μL)	788 [664–1048]	–	
Current CD4/CD8 T-cell ratio	0.95 [0.67–1.10]	–	
Duration of CD4+ T-cell count ≥350 cells/μL, y	11.0 [7.9–14.3]	–	
Current HIV plVL (log_10_)	1.0 [1.0–1.0]	–	
Highest HIV plVL (log_10_)	4.9 [4.3–5.4]	–	
Duration of aviremia, y	11.4 [7.8–13.6]	–	
Duration of aviremia & CD4+ T-cell count ≥350 cells/μL, y	10.8 [7.8–13.0]	–	
Lipodystrophy, yes/no	29/23	–	

^a^ Values are median [interquartile range], unless stated otherwise.

^b^ Mann-Whitney U-test.

^c^ Chi^2^ test.

^d^ Fisher’s exact-test.

PLHIVs: human immunodeficiency virus-infected participants; HUCs: HIV-uninfected controls; HIV: human immunodeficiency virus; CDC: Centers for Disease Control; IVDU: intravenous drug users; MoCA: Montreal Cognitive Assessment; AIDS: acquired immunodeficiency syndrome; plVL: plasma HIV load; hypertension defined as a systolic BP ≥140 mm Hg, diastolic BP ≥90 mm Hg, or the combination of self-reported high BP diagnosis and the use of anti-hypertensive medications; MRI: magnetic resonance imaging; NR: not reported.

The median CD4+ T-cell nadir was 249 cells/μL and median CD4/CD8 T-cell ratio was 0.95. All PLHIVs had plVLs <20 copies/mL for 11±4 years and achieved immune recovery on cART, including aviremia and CD4+ T-cell counts >350 cells/μL for 10±3 years.

pRNFL thicknesses, total retinal macular volumes and GCL macular volumes and thicknesses (Table 3) did not differ between PLHIVs and HUCs. These analyses were repeated using the other eye when both eyes were assessable and the results were comparable (S1 Table). Potential confounding factors (axial length, spherical equivalent and Optical Quality Analyzing System (OQAS)-assessed media opacity) were comparable for PLHIVs and HUCs.

**Table 3:** Spectral domain-optical coherence tomography or visual function measurements and ocular findings of PLHIV and HUC participants.

Parameter	PLHIVs (n = 56)	HUCs (n = 56)	p value
Structural, mean±SD			
pRNFL thickness, (μm)			
Overall pRNFL^a^	99.5±9.6	99.6±8.3	0.97^b^
Nasal	75.9±15.2	75.6±15.2	0.82^c^
Inferior	128.9±14.5	130.2±14.5	
Temporal	70.7±11.5	68.9±11.5	
Superior	121.4±14.8	123.8±14.8	
Macula, mean±SD			
EDTRS total macular volume (mm^3^)	8.7±0.4	8.6±0.4	0.52^b^
EDTRS GCL volume (mm^3^)	0.956± 0.099	0.936±0.115	0.32^b^
EDTRS GCL thickness (μm)			
Fovea	17.6±6.6	15.8±6.6	0.31^c^
Parafovea superior	48.3±8.0	46.5±8.0	
Parafovea temporal	48.5±8.1	47.0±8.1	
Parafovea inferior	49.2±8.6	47.8±8.6	
Parafovea nasal	46.5±9.1	46.7±9.1	
Perifovea superior	33.3±4.3	32.7±4.3	
Perifovea temporal	28.7±3.2	27.9±3.2	
Perifovea inferior	31.1±4.1	30.6±4.1	
Perifovea nasal	27.4±3.0	27.2±3.0	
Choroidal thickness (μm)	330±99	306±96	0.19^b^
Functional, mean±SD			
High-contrast VA EDTRS score	87.1±4.5	87.7±5.2	0.5^b^
2.5% low-contrast VA EDTRS score	22.8±6.6	24.2±8.6	0.34^b^
VF foveal threshold (dB)	37.0±1.7	37.1±1.5	0.73^b^
VF mean deviation (dB)	–1.34±1.68	–0.33±1.46	**<0.001**^b^
VF pattern standard deviation (dB)	2.02±0.98	1.84±0.64	0.26^b^
Color vision (total errors score)	16.37±2.71	16.41±4.86	0.96^b^
Color vision (C-index)	1.59±0.31	1.57±0.52	0.87^b^
Ocular features, mean±SD			
Axial length (mm)	23.75±0.83	23.82±0.74	0.66^b^
Spherical equivalent refraction (diopter)	0.35±1.03	0.14±1.02	0.27^b^
Objective scattering index	0.77±0.52	0.82±0.73	0.73^b^
Intraocular pressure (mm Hg)	13.9±2.8	15.1±2.8	**0.02**^b^

^a^ Overall pRNFL thickness is the average of the four quadrants.

^b^ Mann-Whitney U-test.

^c^ Between-group [PLHIVs vs. HUCs] ANOVA on repeated measures (pRNFL quadrants and GCL ETDRS-grid subfields). PLHIVs: human immunodeficiency virus-infected participants; HUCs: HIV-uninfected controls; HIV: human immunodeficiency virus; pRNFL: peripapillary retinal nerve-fiber layer; SD: standard deviation; EDTRS: Early Treatment Diabetic Retinopathy Study; GCL: ganglion-cell layer; VA: visual acuity; dB: decibels; VF: visual field; C-index: color confusion index; mm Hg: millimeters of mercury.

Among the functional parameters (Table 3), high contrast VA, low contrast VA and color vision were comparable for PLHIVs and HUCs. However, visual field mean deviations (MDs) and intraocular pressure (IOP) differed significantly, being slightly lower for PLHIVs than HUCs but still within normal limits.

**Fig 1:**
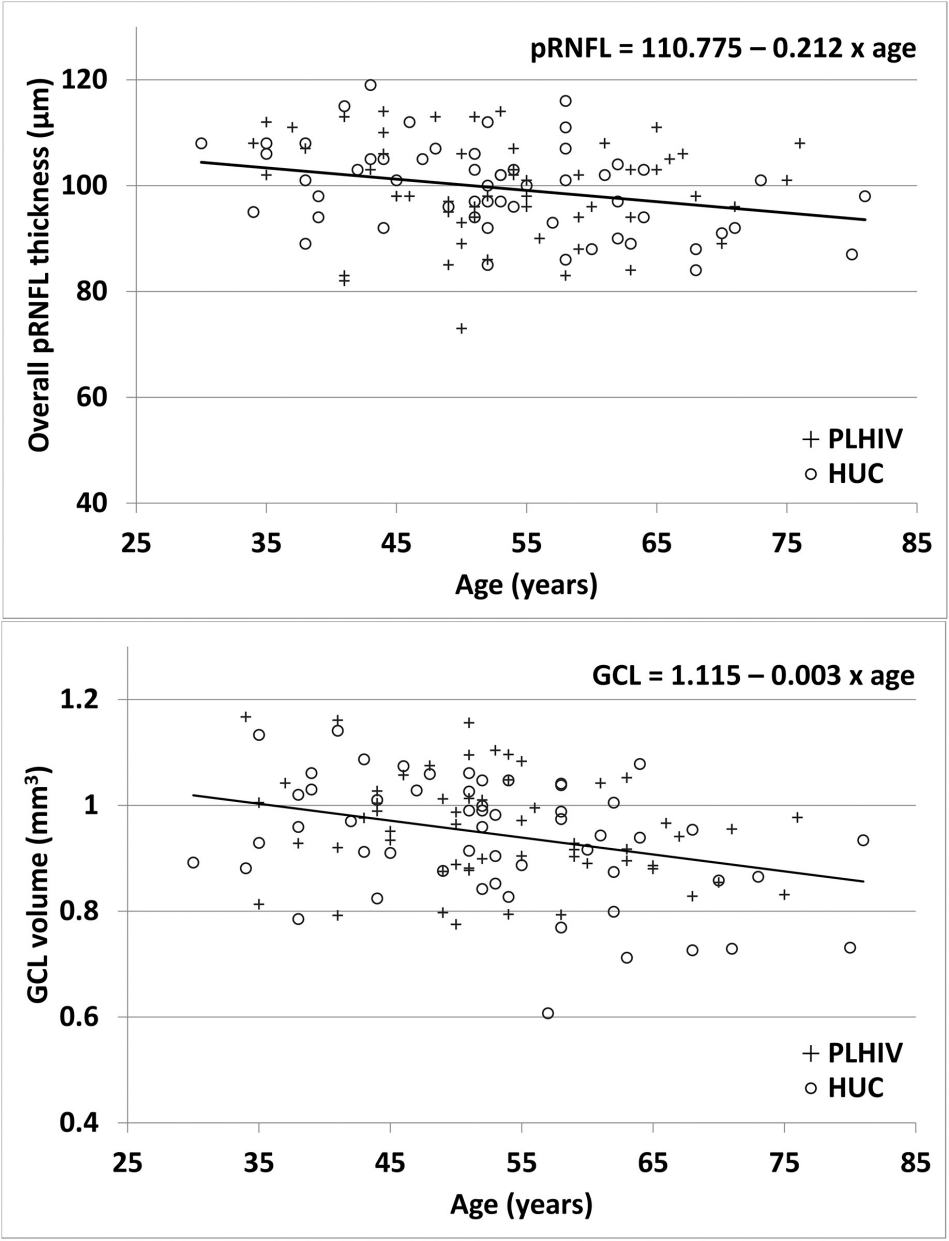
Association between overall peripapillary retinal nerve-fiber–layer (pRNFL) thickness (top) or ganglion-cell–layer (GCL) volume (bottom) and age of PLHIVs and HUCs. Significant linear correlations were found between the overall pRNFL or macular GCL volume and the ages of the persons living with human immunodeficiency virus (PLHIVs) or the HIV-uninfected controls (HUCs). The HIV status had no significant effect on this association. The linear-regression equation is given.

**Fig 2:**
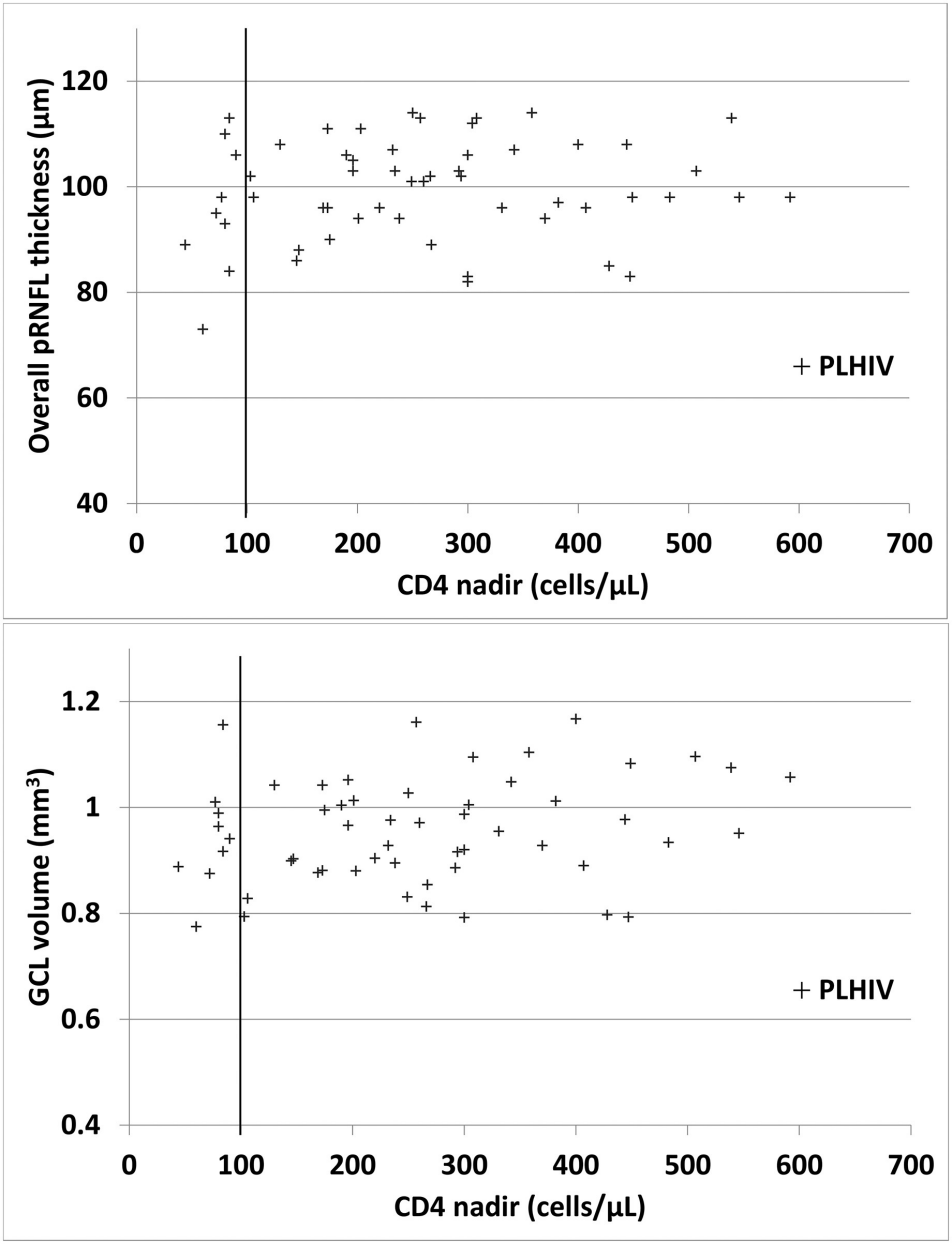
No association between PLHIVs’ overall peripapillary retinal nerve-fiber–layer (pRNFL) thickness (top) or ganglion-cell–layer (GCL) volume (bottom) and CD4+ T-cell count nadirs of the persons living with human immunodeficiency virus (PLHIVs). The vertical line represents the CD4+ T-cell count nadir of 100 cells/μL, because previous studies found that only PLHIVs with nadirs <100 cells/μL were more likely to have thinner pRNFLs. In our study only nine PLHIVs had a nadir <100 cells/μL but their pRNFL thicknesses and macular GCL volumes did not differ from those of the other PLHIVs.

Associations between structural measures and other variables are reported in Table 4. Age was associated with overall pRNFL thickness, GCL macular volume and total retinal macular volume, with HIV status having no significant effect (Fig 1). Among the PLHIVs, no significant association between structural measures and HIV-infection duration or CD4+ T-cell nadir was found (Table 4, Fig 2).

**Table 4:** Associations between structural measurements and other variables of PLHIVs and/or HUCs.

	Outcome variables							
Analysis (predictor variables)	Overall pRNFL thickness^a^		Temporal pRNFL thickness		Total retina macular volume		GCL macular volume	
	β	p	β	p	β	p	β	p
PLHIVs and HUCs (HIV+/–)								
High-contrast VA EDTRS score^b^	**0.43 μm/letter**	**0.01**	0.13 μm/letter	0.57	**0.02 mm**^**3**^**/letter**	**0.04**	**0.004 mm**^**3**^**/letter**	**0.03**
	HIV+/–interaction	0.84	HIV+/–interaction	0.38	HIV+/–interaction	0.41	HIV+/–interaction	0.21
2.5% low-contrast VA EDTRS score^b^	0.10 μm/letter	0.36	0.18 μm/letter	0.21	0.01 mm^3^/letter	0.07	0.002 mm^3^/letter	0.13
	HIV+/–interaction	0.97	HIV+/–interaction	0.30	HIV+/–interaction	0.34	HIV+/–interaction	0.17
VF mean deviation^b^	0.78 μm/dB	0.15	0.37 μm/dB	0.60	0.00 mm^3^/dB	0.93	–0.001 mm^3^/dB	0.83
	HIV+/–interaction	0.68	HIV+/–interaction	0.33	HIV+/–interaction	0.53	HIV+/–interaction	0.38
Color vision C-index^b^	0.4 5 μm/U	0.82	0.69 μm/U	0.79	–0.04 mm^3^/U	0.69	0.020 mm^3^/U	0.41
	HIV+/–interaction	0.97	HIV+/–interaction	0.59	HIV+/–interaction	0.47	HIV+/–interaction	0.36
Axial length^b^	**–4.45 μm/mm**	**<0.001**	1.55 μm/mm	0.27	**–0.12 mm**^**3**^**/mm**	**0.02**	–0.021 mm^3^/mm	0.11
	HIV+/–interaction	0.83	HIV+/–interaction	0.37	HIV+/–interaction	0.55	HIV+/–interaction	0.35
Age^b^	**–0.21 μm/year**	**0.007**	–0.14 μm/year	0.16	**–0.02 mm**^**3**^**/year**	**<0.001**	**–0.003 mm**^**3**^**/year**	**<0.001**
	HIV+/–interaction	0.99	HIV+/–interaction	0.38	HIV+/–interaction	0.44	HIV+/–interaction	0.28
PLHIVs								
Duration of HIV seropositivity^c^	–0.46 μm/year	0.07	–0.28 μm/year	0.33	–0.01 mm^3^/year	0.23	–0.002 mm^3^/year	0.16
CD4+ T-cell count nadir, cells/μL^d^	0.08 μm/(cells/μL)^e^	0.40	0.001 μm/(cells/μL)^e^	0.90	0.00 mm^3^/(cells/μL)	0.25	0.000 mm^3^/(cells/μL)	0.09

^a^ Overall pRNFL thickness is the average of the four quadrants.

^b^ Linear-regression models including HIV status.

^c^ Multivariable linear-regression analysis using age and duration of HIV seropositivity.

^d^ Univariate linear-regression analysis.

^e^ Thickness unit/(nadir unit in cells/μL).

PLHIVs: persons living with human immunodeficiency virus-infected; HUCs: HIV-uninfected controls; HIV: human immunodeficiency virus; pRNFL: peripapillary retinal nerve-fiber layer; EDTRS: Early Treatment Diabetic Retinopathy Study; GCL: ganglion-cell layer; VA: visual acuity; dB: decibels; VF: visual field; C-index: color confusion index.

**Fig 3:**
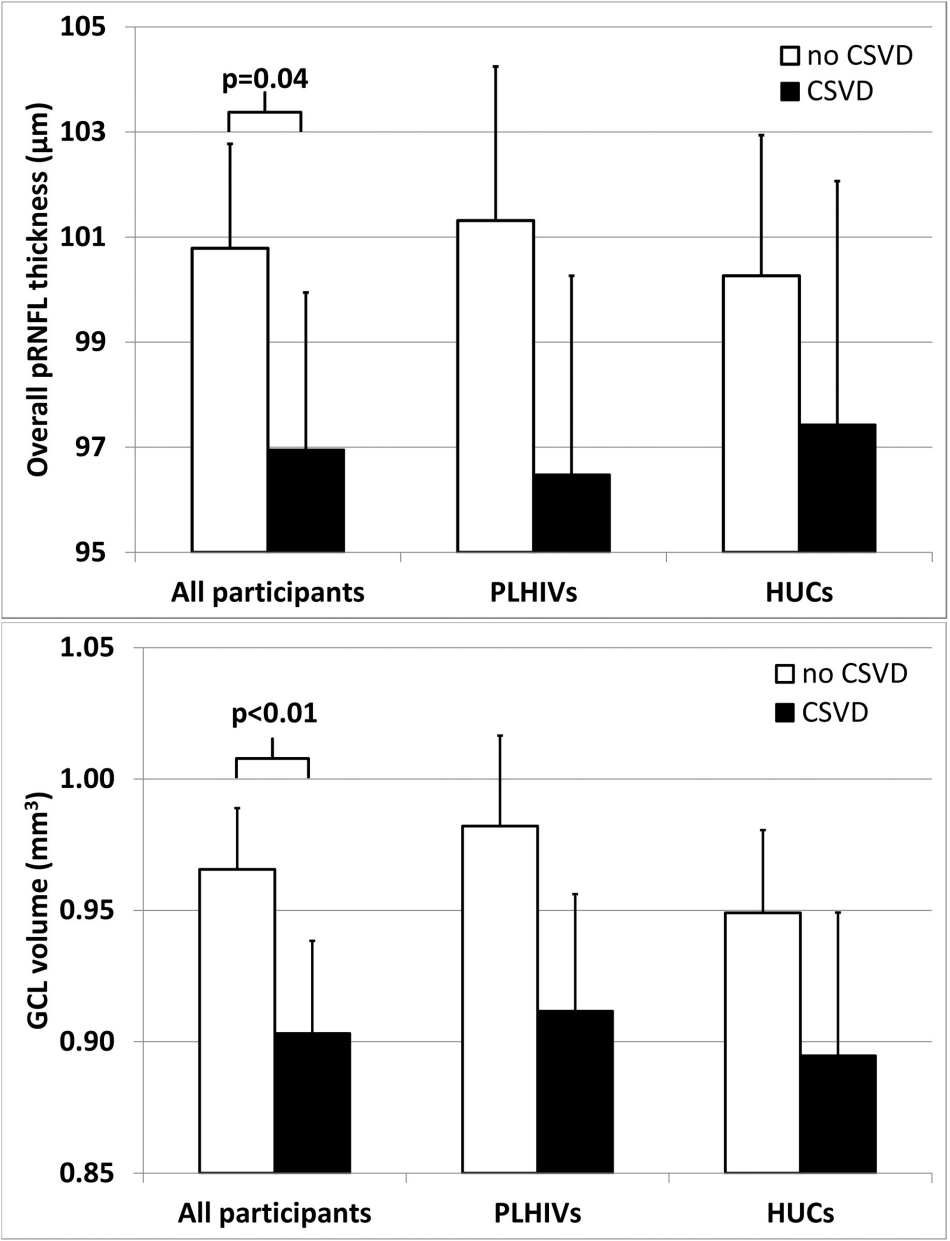
Effect of cerebral small-vessel disease (CSVD) on overall peripapillary retinal nerve-fiber–layer (pRNFL) thickness (top) and ganglion-cell–layer (GCL) volume (bottom) in all participants. The mean overall pRNFL thickness (top) and the mean macular GCL volume (bottom) are reported for all participants, persons living with human immunodeficiency virus (PLHIVs) and for HIV-uninfected controls (HUCs). Error bars represent the standard deviation. Participants with MRI-defined CSVD had significantly thinner pRNFL (p = 0.04; ANOVA) and smaller macular GCL volume (p<0.01; ANOVA) compared to the participants with no CSVD. HIV status had no significant effect on pRNFL and no significant interaction with the effect of CSVD on pRNFL (S2 Table).

MRIs did not reveal a lesion that could cause optic neuropathy or trans-synaptic retrograde degeneration within the optic nerves. MRI detected CSVD in 21 (38%) PLHIVs and 14 (25%) HUCs (p = 0.15); it mainly reflected WMHs of presumed vascular origin (21 PLHIVs versus 12 HUCs, p = 0.06). Documented CSVD was associated with overall thinner pRNFLs, smaller whole retinal macular volumes and smaller GCL macular volumes for all participants, with HIV status having no significant effect (Fig 3, S1 Table).

## Discussion

Our results showed that overall and 4-quadrant (localized) pRNFLs, total retinal macular volumes, GCL macular volumes and EDTRS-grid–defined GCL regional thicknesses were not smaller in long-term–sustained, immunovirologically controlled PLHIVs compared to age- and sex-matched HUCs.

Our results agree with those of two studies [5,24] that had included long-term, cART-treated, immunovirologically well-controlled HIV+ individuals. However, our results (versus [5] or [24], respectively) extend their findings because our population is more homogenous, facilitating exploration of the impact of: (1) longer-known HIV infection (median 20 [range 11–30] versus median 15 [range 1–27] or mean 15 [range 1–30] years), (2) longer cART exposure (median 17 [range 6–22] versus median 12 [range 1–21] years or unavailable), (3) more prolonged plVL undetectability (median 11 [range 5–17] versus median 10 [range 0–15] years or unavailable), (4) less severe immunosuppression (CD4+ T-count nadir median 249 [range 158–350] versus median 180 [range 0–620] and mean 172 [range 1–552] cells/μL) and (5) well-sustained immunovirological variables (duration of plVL undetectability and CD4+ T-cell counts >350 μL (median 11 [range 5–17] years versus unavailable for both studies; CD4/CD8 T-cell ratio (median 0.95 [range 0.67–1.10] versus median 0.75 [range 0.29–4.13] or unavailable).

According to Invernizzi et al. [24], the GCL was thinner only for the subgroup of cART-treated PLHIVs with a mean MoCA score <26/30 (n = 34) compared to HUCs. For PLHIVs with a MoCA score ≥26/30 (n = 35), GCL thickness was comparable to that of HUCs, as we found. As for Invernizzi et al. [24], the MoCA score was neither an inclusion nor exclusion criterion for our study. Only one of our PLHIVs had a MoCA score <26/30 (i.e., 25/30), probably because they met the parameters associated with the most preserved cognitive functions, i.e.: high educational level, CD4+ T-cell count nadir >200 cells/μL and current CD4/CD8 T-cell ratio ~1 [35–41].

Significant overall pRNFL thinning was found in other studies, but affected only PLHIVs with CD4+ T-cell count nadirs <100 cells/μL for ≥6 months, compared to PLHIVs with nadirs >100 cells/μL [21,22] or HUCs [21,23]. However, many HIV variables were missing in those reports: (1) HIV-seropositivity duration [22,23,42–44], (2) cART duration [22–24,44,45], (3) current plVL or duration of undetectability [21–23,42–43,46] and (4) current CD4+ T-cell counts [22,23,43,45]. Hence, those studies’ results cannot explain whether the pRNFL thinning could be attributed to severe immunodeficiency alone or its combination with prolonged HIV infection without sustained immunovirological control.

The higher frequency of CVSD-surrogate WMHs in aviremic, cART-treated PLHIVs compared to HUCs, not related to any ART classes, was recently reported [10,47]. We found only a trend toward significance for WMHs of presumed vascular origin between PLHIVs and HUC (p = 0.06). That failure to reach significance is probably due to a lack of statistical power of our study. These vascular abnormalities were associated with thinner overall pRNFLs, smaller total retina macular volumes and smaller CGL macular volumes, independently of HIV status. A pRNFL thinning or defect was reported previously for the arteriosclerotic CSVD form [26] and cerebral autosomal-dominant arteriopathy with subcortical infarcts and leukoencephalopathy (CADASIL) [48], a genetic form of CSVD. To the best of our knowledge, that association has not been reported previously in adult, cART-treated PLHIVs with CSVD. CSVD is characterized by thickening of the walls of the small perforating arteries in the brain, resulting in low cerebral blood flow [49]. A causative role of cerebral hypoperfusion and decreased perfusion of the inner retinal layers has been advanced to explain the association of CSVD with pRNFL and GCL thinning, as shown for CADASIL [50]. Our results agree with an emerging change of the NeuroHIV paradigm, highlighting the potential contribution of vascular brain damage in aging PLHIVs [16].

Despite being the first combined OCT–MRI study in middle-aged, cART-treated PLHIVs with well-sustained immune restoration, our study might suffer from a lack statistical power. Although our study-sample size might be considered relatively small, it is in accordance with previous similar publications (Table 1) and is sufficient to highlight pRNFL differences in these subjects. Although PLHIV and HUC pRNFL-thickness sameness cannot be established, our study’s statistical power to detect thinning of –5 μm was 90%. Interestingly, the test–retest variability of SD-OCT–measured pRNFL thickness was ~5 μm [51]. Because the primary endpoint and the power calculation were based on the overall pRNFL thickness, interpretation of the lack of a smaller GCL volume and localized pRNFL thinning in our study requires prudence. Localized pRNFL thinning could result from methodological biases secondary to the multiplicity of statistical comparisons without control for inflation of the type-1 error [52]. That bias was also seen in macula studies, in which multiple quadrants and multiple retinal layer thicknesses generated high numbers of p values. Such methodological biases were previously highlighted by Demirkaya et al. [5].

Although our series is not representative of the entire HIV+ population, it is representative of PLHIVs receiving care in northern Europe, where >90% are successfully treated [53]. However, the 2019 UNAIDS world epidemiological data showed that 79% of PLHIVs are aware of their seropositivity, 78% of PLHIVs knowing their HIV status are cART-treated and 86% of those cART-treated PLHIVs have a plVL below the detection threshold (unaids.org). Moreover, it was recently demonstrated that low plVLs of 51–200 copies/mL were strongly associated with virological failure [54]. Thus, HIV-induced brain damage may be more a legacy effect resulting from prior incomplete virus control [54,55]. Those findings provide support for the European definition of virological failure as persistent plVLs of >50 copies/mL (eacsociety.org). Our findings should not be compared or held as a contradiction to historical studies and may better apply to future PLHIV cohorts, for whom the therapeutic guidelines recommend that cART must be initiated for a CD4+ T-cell count threshold ≥500 cells/μL [56]. Indeed, the CASCADE study showed that PLHIVs are mostly diagnosed and treated with a CD4+ T-cell count nadir >200 cells/μL [57]. Recent findings showed that full viral suppression may preserve long-term PLHIVs’ brain health [58]. Hence, continuing to report results concerning virologically uncontrolled, cART-treated PLHIVs is not really suitable [54,55,58].

In addition to the OCT evaluation of visual pathway structures, most visual evaluation and ocular parameters were similar for PLHIVs and HUCs, with the exception of visual field MDs and IOP. Decreased visual field MD is a classic sign of HIV–NRD but in our study it cannot be explained by pRNFL thinning or decreased GCL macular volume. That decline could however be explained by functional changes without structural damage of the retinal ganglion cells or other cells implicated in vision. Indeed, decreased cone-photoreceptor density in HIV+ participants was found using an adaptive optics camera [59] and SD-OCT visualized possible changes in retinal layers other than GCL and RNFL [5,60,61]. Lower IOP in HIV+ participants was previously described and its causes are more likely multifactorial [61]. Our results showed that this diminished IOP persists in PLHIVs with long-term, well-sustained immune control.

Our results showed that cART-treated PLHIVs who successfully achieved sustained immunovirological control, even with HIV infection lasting 20 years, do not have pRNFL thinning. That is an optimistic finding in terms of PLHIVs aging. However, in line with recent results from a study on HIV-infected children [28], as in the general population, CSVD in our middle-aged PLHIVs was associated with thinner pRNFLs and GCLs.

## Supporting information

S1 Fig The 9 Early Treatment Diabetic Retinopathy Study (ETDRS)-grid subfields used to analyze ganglion-cell layer thickness in right and left eyes. 1, fovea; 2, parafovea superior; 3, parafovea temporal; 4, parafovea inferior; 5, parafovea nasal; 6, perifovea superior; 7, perifovea temporal; 8, perifovea inferior; and 9, perifovea nasal.
(JPG)

S1 Table Associations between radiological and spectral-domain optical coherence tomography findings and HIV status. (DOCX)

S2 Table Optical coherence tomography measurements in PLHIVs and HUCs using the other eye when both eyes were assessable. PLHIVs, persons living with HIV: HUCs, healthy uninfected controls.
(DOCX)

S1 File Excel file containing all the relevant data underlying the findings described in the manuscript. (XLSX)
