# Correction: Absence of peripapillary retinal nerve-fiber–layer thinning in combined antiretroviral therapy-treated, well-sustained aviremic persons living with HIV

**DOI:** 10.1371/journal.pone.0234497

**Published:** 2020-06-04

**Authors:** Cedric Lamirel, Nadia Valin, Julien Savatovsky, François-Xavier Lescure, Anne-Sophie Alonso, Philippe Girard, Jean-Paul Vincensini, Pierre-Marie Girard, Laurence Salomon, Isabelle Cochereau, Antoine Moulignier

There are errors in the Competing Interests statement. The correct Competing Interests statement is as follows: C. Lamirel, N. Valin, J. Savatosky, A.-S. Alonso, J.-P. Vincensini, L. Salomon and I. Cochereau have no disclosures to report. F.-X. Lescure has received funding for lectures from Gilead Sciences and MSD France; and to travel to meetings from MSD France, Astellas and Eumedica. P. Girard has received support to travel to meetings and accommodations from Bayer and Leo Pharma. P.-M. Girard has received honoraria for participation on international advisory boards from Gilead, ViiV Healthcare and Abbvie, and honoraria for speaking engagements from Janssen and BMS. A. Moulignier has received research support from the Association Nationale de Recherche sur le SIDA (ANRS), funding for lectures from Biogen Idec and Norvartis, and to travel to meetings from Biogen Idec and Teva Pharma. This does not alter our adherence to PLOS ONE policies on sharing data and materials. There are no patents, products in development or marketed products associated with this research to declare.
