# Supplementary figures and images for: Absence of peripapillary retinal nerve-fiber–layer thinning in combined antiretroviral therapy-treated, well-sustained aviremic persons living with HIV
Source: PLoS One. 2020 Mar 10;15(3):e0229977. doi: 10.1371/journal.pone.0229977 (PMC7064175; doi:10.1371/journal.pone.0229977)

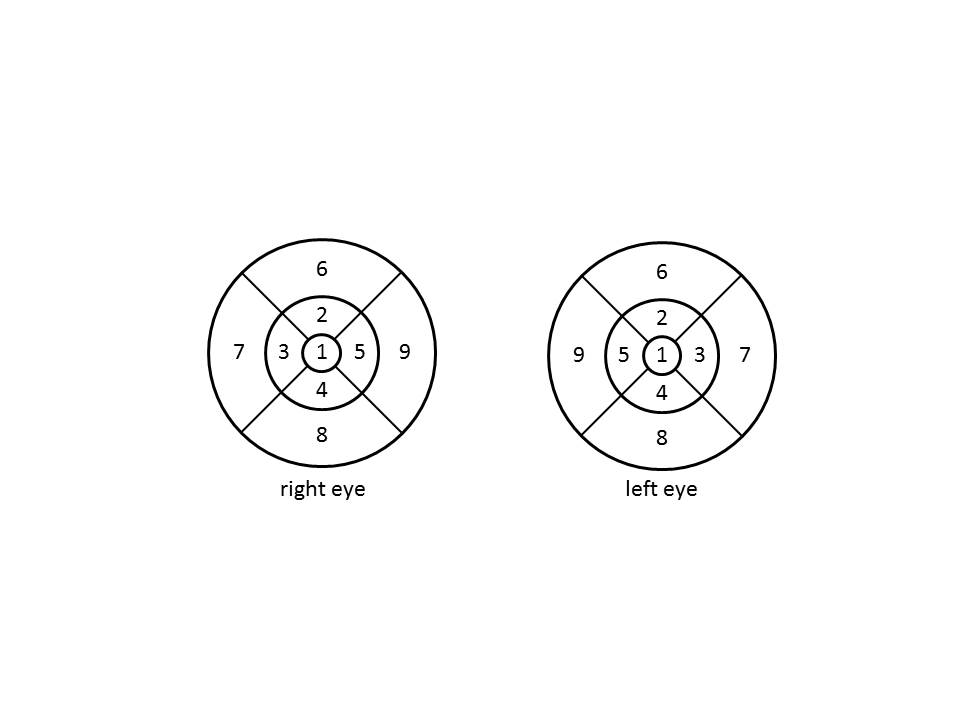

Supplement: S1 Fig — 1, fovea; 2, parafovea superior; 3, parafovea temporal; 4, parafovea inferior; 5, parafovea nasal; 6, perifovea superior; 7, perifovea temporal; 8, perifovea inferior; and 9, perifovea nasal. (JPG) [file pone.0229977.s001.jpg]
