# Supplementary material for: Absence of peripapillary retinal nerve-fiber–layer thinning in combined antiretroviral therapy-treated, well-sustained aviremic persons living with HIV
Source: PLoS One. 2020 Mar 10;15(3):e0229977. doi: 10.1371/journal.pone.0229977 (PMC7064175; doi:10.1371/journal.pone.0229977)
Supplement: S1 Table — (DOCX) [file pone.0229977.s002.docx]

**S1 Table. Spectral-domain optical coherence tomography measurements in PLHIVs and HUCs using the other eye when both eyes were assessable**

| Variable | PLHIVs | HUCs | p value |
| --- | --- | --- | --- |
| Structural, mean±SD |  |  |  |
| pRNFL thickness, (µm) |  |  |  |
| Overall pRNFL^a^ | 98.8±9.7 | 99.5±8.4 | 0.66^b^ |
| Nasal | 74.5±15.1 | 75.4±15.1 | 0.50^c^ |
| Inferior | 129.0±14.3 | 129.4±14.3 |  |
| Temporal | 70.2±11.2 | 68.4±11.2 |  |
| Superior | 120.1±15.1 | 125.2±15.1 |  |
| Macula |  |  |  |
| EDTRS total macular volume (mm^3^) | 8.7±0.4 | 8.6±0.4 | 0.32^b^ |
| EDTRS GCL volume (mm^3^) | 0.956±0.104 | 0.932± 0.118 | 0.25^b^ |
| EDTRS GCL thickness (µm) |  |  |  |
| Fovea | 17.8±6.7 | 16.1±6.7 | 0.25^c^ |
| Parafovea superior | 48.3±8.4 | 46.4±8.4 |  |
| Parafovea temporal | 48.4±8.3 | 47.5±8.3 |  |
| Parafovea inferior | 49.2±9.2 | 48.0±9.2 |  |
| Parafovea nasal | 48.2±9.4 | 46.4±9.4 |  |
| Perifovea superior | 32.6±4.3 | 31.9±4.3 |  |
| Perifovea temporal | 28.6±3.3 | 27.7± 3.3 |  |
| Perifovea inferior | 31.5±4.2 | 31.0±4.2 |  |
| Perifovea nasal | 27.5±3.0 | 27.0±3.0 |  |
| Choroidal thickness (µm) | 318±99 | 304±92 | 0.19^b^ |

^a^ Overall pRNFL thickness is the average of the four quadrants.

^b^ Mann-Whitney U-test.

^c^ Between-group [PLHIVs vs. HUCs] ANOVA on repeated measures (pRNFL quadrants and GCL ETDRS-grid subfields).

PLHIV: persons living with human immunodeficiency virus-infected; HUCs: HIV-uninfected controls; pRNFL: peripapillary retinal nerve-fiber layer; SD: standard deviation; ANOVA: analysis of variance; EDTRS: Early Treatment Diabetic Retinopathy Study; GCL: ganglion-cell layer.
