# Supplementary material for: Absence of peripapillary retinal nerve-fiber–layer thinning in combined antiretroviral therapy-treated, well-sustained aviremic persons living with HIV
Source: PLoS One. 2020 Mar 10;15(3):e0229977. doi: 10.1371/journal.pone.0229977 (PMC7064175; doi:10.1371/journal.pone.0229977)
Supplement: S2 Table — PLHIVs, persons living with HIV: HUCs, healthy uninfected controls. (DOCX) [file pone.0229977.s003.docx]

**S2 Table. Associations between radiological and spectral-domain optical coherence tomography findings and HIV status**

|  | Overall pRNFL thickness^a^ |  | Temporal pRNFL thickness^a^ |  | Total retina macular volume^a^ |  | GCL volume^a^ |
| --- | --- | --- | --- | --- | --- | --- | --- |
|  | F(1, 108) p value |  | F(1, 108) p value |  | F(1, 108) *P* |  | F(1, 108) p value |
| Effect of HIV | 0.00 .98 |  | 1.90 .17 |  | 0.44 .51 |  | 1.35 25 |
| Effect of CSVD | **4.38 .04*** |  | 3.54 .06 |  | **7.58 <.01*** |  | **8.39 <.01*** |
| HIV–CSVD interaction | 0.30 .59 |  | 0.88 .35 |  | 0.55 .46 |  | 1.35 .25 |

* p<0.05.

^a^ ANOVA with 2 factors: HIV status and presence/absence of MRI-detected CSVD. Overall pRNFL thickness is the average of the four quadrants.

HIV: human immunodeficiency virus; pRNFL: peripapillary retinal nerve-fiber layer; GCL: ganglion-cell layer; CSVD: cerebral small-vessel disease.
